# An Exploratory Study of Consanguinity and Dental Developmental Anomalies

**DOI:** 10.5005/jp-journals-10005-1567

**Published:** 2018

**Authors:** Saima Y Khan

**Affiliations:** Department of Pediatric and Preventive Dentistry, Aligarh Muslim University, Aligarh, Uttar Pradesh, India

**Keywords:** Consanguinity, Fusion, Microdontia, Nonsyndromic supernumerary teeth

## Abstract

**Background:**

Consanguinity is known to alter the population structure but the available literature is almost silent on the association of dental conditions with consanguinity.

**Aim:**

The purpose was to assess the various dental developmental anomalies in 6–9 year old children born out of consanguineous and non-consanguineous marriages and its association with their parents.

**Design:**

A cross sectional house–hold survey with a sample size of 2,000 (1,600 non-consanguineous and 400 consanguineous respondents and their parents) using systematic random sampling was planned. Six to nine-year-old children and their parents living in 1,597 households were examined and the information recorded on a pretested self prepared questionnaire. The questionnaire had questions pertaining to personal details, type of consanguineous marriages, history of trauma and examination of dental developmental anomalies.

**Results:**

Multivariate logistic regression showed that non syndromic supernumerary teeth in fathers (*p* =.009); fusion in mothers (*p* = 0.002); fusion (*p* <0.001), nonsyndromic supernumerary teeth (*p* < 0.001), and microdontia (*p* = 0.002) in respondents were significantly associated with consanguinity.

**Conclusion:**

A significant association of developmental anomalies in parents with consanguineous marriages and their respondents was observed.

**How to cite this article:**

Khan SY. An Exploratory Study of Consanguinity and Dental Developmental Anomalies. Int J Clin Pediatr Dent, 2018;11(6):513-518

## INTRODUCTION

Linguistically, consanguinity is a term derived from two Latin words “con” meaning common or of the same and “sanguineus” meaning blood; hence, referring to a relationship between individuals of the same blood. Consanguinity in a way is responsible for alteration of genotypic frequencies; hence, influences the structure and formation of a population^1^, as a carrier is unlikely to find a partner who carries the same disorder unless they are related. Literature is available since ages on the association of medical conditions like blood dyscrasias, and mental conditions with consanguinity; however, the literature is almost silent on the association of dental conditions with consanguinity.

As far as India is concerned, till date to the best of my knowledge, no such study exploring the true association of dental developmental anomalies in parents with consanguineous marriage and their respondents and consanguinity has been carried out. Thus, this issue becomes very important, and that is why the present study was planned.

## MATERIALS AND METHODS

### Type and Design of the Study

A household survey using a cross-sectional study design was planned.

### Setting

A community-based approach was used after obtaining the sample size; the researcher conducted the study by visiting every tenth household of every 10th ward of Aligarh city, Uttar Pradesh, India. The information was recorded on a meticulously self-prepared and pretested questionnaire, which was used to examine the respondent and their parents, respectively.

### Study Population

**Fig. 1:**
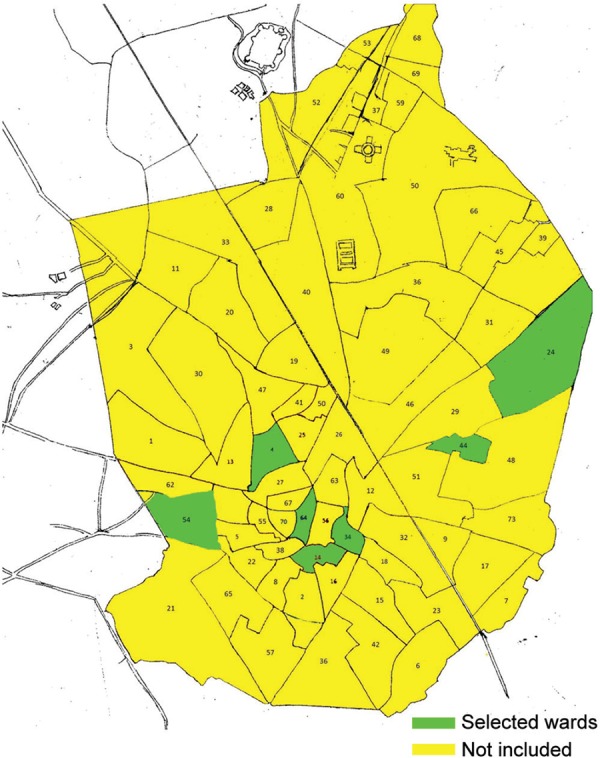
Map of 70 wards

The study population included the children aged 6–9 years (1,600 nonconsanguineous and 400 consanguineous) and their parents living in 1597 households in seven selected wards of Aligarh city, India (Fig. 1).

### Sampling Frame

The sampling frame was bound by the following inclusion and exclusion criteria.

### Inclusion Criteria

- Children aged 6–9 years.
- Permanent resident of Aligarh city living permanently in Aligarh since birth.
- Healthy children.
- Both parents alive.

### Exclusion Criteria

- Children living continuously outside Aligarh for a duration exceeding six months ever since their birth.
- Nonhealthy children.
- Premature births.
- Children whose mothers were exposed to radiation during pregnancy.
- Children whose mothers had taken vaccination against rubella/varicella during pregnancy.
- Children whose mothers were on long term medication during pregnancy.
- All those not willing to participate in the study.

### Sampling Method

Multilayered sampling method (stratified random sampling) was used. In the first layer, it was assumed that the prevalence of consanguinity itself is around 20% in the study population. To reject the hypothesis that the prevalence of dental developmental anomalies is different in children born to consanguineous parents, we required the sample size five times higher than the calculated sample size to precisely reach an equal number of children born to consanguineous parents.

The sample size was calculated using the following formula:

**Figure:**
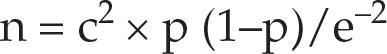


### Assessment of Age of the Child

Majority of the parents produced the birth certificates in support of age of the child. In a few cases, the age was determined in relation to the festivals (commonly used age determination pattern in India). Dentition at a particular age also acted as a supportive adjunct.

### Permission and Clearances

Permission to carry out the study was obtained from the Institutional Ethics and Research Advisory Committee, Faculty of Medicine, Aligarh Muslim University, Aligarh, Uttar Pradesh, India (D. No. 41/FM/04/08/15). Informed consent was obtained from all the parents of the respondents, and they were assured of the confidentiality of the information given by them.

### Tool for Data Collection

To conduct a pilot study and to remove intraobserver bias, the information was recorded on a predesigned questionnaire which was administered to 20 children. These 20 children were not included in the study sample. The study was conducted by a single examiner. Standardization and validity of the observer were done before the conduct of the study. The mean Kappa value was found to be 0.86. The overall internal reliability of the questionnaire was 0.74 according to Cronbach's alpha. After testing and making the necessary corrections in the questionnaire used in the pilot study, the respondents and their parents were interviewed and recorded on a self-prepared and pretested questionnaire.

### Statistical Analysis

Questionnaires were coded, and data analysis was done by employing statistical package for social sciences (SPSS) version 16 software. Descriptive statistics (frequency and percentages) were applied. Chi-square test of significance and Fisher's exact test was used for comparison between categorical variables. A significant difference was considered at *p* <0.05 and 95% confidence interval. Multivariate logistic regression analysis was performed with consanguinity as dependent variable and religion, history of trauma, and developmental anomalies as independent variables.

**Table 1:** Distribution of personal characteristics, type of consanguineous marriages, and history of trauma in consanguineous and nonconsanguineous group

*Personal characteristics*	*Nonconsanguineous* *(n =1600)*		*Consanguineous* *(n = 400)*		*Fisher's exact test/pearson chi-square*		*p value*
	*No.*	*%*	*No.*	*%*			
Age of respondents (years)							
6	450	28.1%	68	17.0%	29.76	3	0.000*
7	380	23.8%	136	34.0%			
8	506	31.6%	120	30.0%			
9	264	16.5%	76	19.0%			
Gender of respondents							
Female	608	38%	124	31%	67.57	1	0.009*
Male	992	62%	276	69%			
Religion of respondents							
Hindu	1152	72%	0	0%	694.2	4	0.008*
Islam	420	26.20%	386	96.5%			
Christian	16	1%	6	1.50%			
Buddhist	6	0.40%	8	2%			
Sikh	6	0.40%	0	0%			
Type of consanguineous marriage							
First cousin	−	−	340	85%	N.A	N.A	N.A
Second cousin	−	−	26	6.5%			
Third Cousin	−	−	34	8.5%			
History of trauma							
Mother	1523	95.2%	388	97%	Fisher's Exact test		
	77	4.8%	12	3.0%			
Father	1540	96.2%	390	97.5%	1.48	1	
	60	3.8%	10	2.5%			
Respondent	1558	97.4%	396	99%	Fisher's Exact test		
	42	2.6%	4	1.0%			

*Figures in bold depict statistically significant values; *p*: Value of probability

## RESULTS

Table 1 shows that the highest numbers of respondents in nonconsanguineous group were 506 (31.6%) 8-year-old and 136 (34%) 7-year-old in the consanguineous group. The difference was statistically highly significant (*p* <0.001). Males outnumbered the females in both the study groups, i.e., 992 males (62%) in the nonconsanguineous group and 276 males (69%) in the consanguineous group. Difference was statistically significant (*p* = 0.009). By religion, the majority of respondents in the nonconsanguineous group were hindus 1,152 (72%) whereas 386 (96.5%) muslims were in majority in the consanguineous group. The difference was statistically highly significant (*p* <0.001). Three hundred forty (85%) marriages were performed between first cousins and 34 (8.5%) between third cousins, respectively. History of trauma in mother (*p* = 0.071), father (*p* = 0.223) and respondent (*p* = 0.061) was not significant and did not show any association with either of the groups. Table 2 shows the distribution of developmental anomalies in mother, father, and respondent. In mothers: fusion (*p* <0.001), nonsyndromic teeth (*p* <0.001), microdontia (*p* = 0.002), in fathers: fusion (*p* <0.001), gemination (*p* <0.001) and in respondents: fusion (*p* <0.001), gemination (*p* <0.001), nonsyndromic supernumerary teeth (*p* <0.001) and microdontia (*p* = 0.002) were statistically significantly higher in consanguineous group than in nonconsanguineous group. Table 3 for multivariate logistic regression analysis showed nonsyndromic supernumerary teeth (*p* = 0.009) (OR = 0.328) (CI= 0.142–0.757) in fathers, fusion (*p* = 0.002) (OR = 0.058) (CI = 0.010–0.345) in mothers, fusion (*p* <0.001) (OR = 0.180) (CI = 0.072–0.454), nonsyndromic supernumerary teeth (*p* <0.001) (OR = 0.151) (CI = 0.078–0.292) and microdontia (*p* = 0.002) (OR = 0.140) (CI = 0.041–0.480) in respondents were significantly associated with consanguinity. The negative (–) B value in parameter estimates of multivariate logistic regression of developmental anomalies, further strengthens the (odds ratio) and hence the association with consanguinity. In religion, Islam (*p* <0.001) (OR = 6.642 *×* 10^8^) (CI = 1.670 *×* 10^8^–2.641 *×*10^9^) and Christians (*p* <0.001) (OR = 1.829 *×* 10^7^) (CI = 1734127.067–1.930 *×* 10^8^) were significantly associated with consanguinity.

**Table 2:** Distribution of dental developmental anomalies in consanguineous and nonconsanguineous group

*Developmental anomalies*		*Nonconsanguineous* *(n =1600)*		*Consanguineous* *(n = 400)*		*Fisher's exact test/pearson chi-square*	*df*	*p value*
		*No.*	*%*	*No.*	*%*			
Mother								
Fusion	No	1598	99.9%	386	96.5%	Fisher's exact test		0.000*
	Yes	2	0.1%	4	3.5%			
Gemination	No	1600	100%	400	100%	N.A	N.A	N.A
	Yes	0	0%	0	0%			
Nonsyndromic supernumerary teeth	No	1600	100%	390	97.5%	Fisher's exact test		0.000*
	Yes	0	0%	10	2.5%			
Microdontia	No	1600	100%	394	98.5%	Fisher's exact test		0.002*
	Yes	0	0%	6	1.5%			
Father								
Fusion	No	1600	100%	392	98%	Fisher's exact test		0.000*
	Yes	0	0%	8	2%			
Gemination	No	1600	100%	396	96%	Fisher's exact test		0.002*
	Yes	0	0%	4	1%			
Nonsyndromic supernumerary teeth	No	1578	98.6%	388	97.0%	Fisher's exact test		0.310
	Yes	22	1.4%	12	3.0%			
Microdontia	No	1592	99.5%	400	100%	Fisher's exact test		0.370
	Yes	8	0.5%	0	0%			
Respondent								
Fusion	No	1586	99.1%	382	95.5%	Fisher's exact test		0.000*
	Yes	14	0.9%	18	4.5%			
Gemination	No	1600	100%	393	98.2%	Fisher's exact test		0.000*
	Yes	0	0%	7	1.8%			
Nonsyndromic supernumerary teeth	No	1578	98.6%	370	92.5%	Fisher's exact test		0.000*
	Yes	22	1.4%	30	7.5%			
Microdontia	No	1594	99.6%	392	98.0%	Fisher's exact test		0.002*
	Yes	6	0.4%	8	1.2%			

*Figures in bold depict statistically significant values; *p*: value of probability

## DISCUSSION

The main reported consequence of inbreeding or consanguineous marriage is the increased risk of transfer of autosomal recessive disorders from one generation to the next, as a carrier is unlikely to find a partner who carries the same disorder unless they are related.*2,3* First cousins have 12.5% of genes in common, so their children may have overall 6.25% homozygous gene loci.*4,5* Thus, offsprings of first cousin consanguineous marriage have an overall risk of 1 in 20 of being affected or malformed as compared to 1 in 40 in the general population. In the present study, Muslims showed a highest frequency of consanguinity. This finding is in agreement with other studies conducted in Lebanon^6^ and Puducherry, India.^7^ Within different religions, there are varied perceptions regarding consanguinity. Like in Christianity, the orthodox churches have banned consanguineous marriages. The Roman Catholic Church requires special permission for first cousin marriage and the protestants allow marriages up to and including first cousins. In Muslims, the uncle-niece union is prohibited. According to Akrami and Osati^8^ and Saadat*,9,10* the relationship of consanguinity with religion is limited; especially in the Muslim religion, where a *Hadith* (recorded pronouncements) of Prophet Mohammad (PBUH) quotes that “Do not marry cousins, as the offsprings may be disabled at birth”, hence discouraging such marriages.

On the contrary, the Prophet married his daughter to his paternal first cousin. Thus, for Muslims, consanguineous marriages could be taken as Sunnah (deeds of the Prophet). In our study, marriages between the first cousins (85%) was the most preferred marriage. A similar observation has been reported by others;*3,6,11,12* however, another study on Chilean population^13^ showed the second cousin as the most preferred consanguineous marriage. The specific pattern of the consanguineous union depends upon the traditional customs and ethnic beliefs. On to developmental anomalies, the present study revealed the real association of nonsyndromic supernumerary teeth, fusion, and microdontia with consanguinity by multivariate logistic regression. With the various stages in the life cycle of the tooth namely–initiation, proliferation, histodifferentiation, morphodifferentiation, and apposition, a strong genetic component is predicted;^14^ however, the interplay of genetic factors remains unknown and is an area of further research. A study conducted in Uttar Pradesh, India^15^ was in agreement to the present study and found that consanguineous marriages can cause craniofacial abnormalities like (malocclusions–15%, nonsyndromic oligodontia–2%, enamel hypoplasia–2%, cleft lip, and palate–15%, respectively), orofacial pigmentations, and other birth defects.

**Table 3:** Multivariate logistic regression analysis; parameter estimates

*CONSa*		*B*	*Std error*	*Wald*	*df*	*Sig*	*Exp (B)*	*95% confidence interval for exp (B)*	
								*Lowerbound*	*Upperbound*
Intercept		186.580	1441.218	0.017	1	0.897	−	−	−
H/O trauma of father	Yes	−0.135	0.848	0.026	1	0.873	0.873	0.166	4.599
	No	0^b^	−	−	0	−	−	−	−
H/O trauma of mother	Yes	0.102	0.764	0.018	1	.894	1.108	0.248	4.951
	No	0^b^	−	−	0	−	−	−	−
Dev. defects of father									
Fusion	No	-12.701	497.559	0.001	1	0.980	3.050E-6	0.000	0^c^
	Yes	0^b^	−	−	0	−	−	−	−
Gemination	No	-16.215	626.505	0.001	1	.979	9.080E-8	0.000	0^c^
	Yes	0^b^	−	−	0	−	−	−	−
Nonsyndromic supernumerary teeth	No	−1.114	0.426	6.839	1	0.009	0.328	0.142	0.757
	Yes	0^b^	−	−	0	−	−	−	−
Microdontia	No	11.353	341.532	0.001	1	0.973	8.526E4	1.651E-286	4.402E295
	Yes	0^b^	−	−	0	−	−	−	−
Dev. fefects of mother									
Fusion	No	−2.846	0.909	9.803	1	0.002	0.058	0.010	0.345
	Yes	0^b^	−	.	0	−	−	−	−
Gemination	No	0^b^	−	.	0	−	−	−	−
	Yes	−17.049	0.000	.	1	−	3.943E-8	3.943E-8	3.943E-8
Nonsyndromic supernumerary teeth	No	0^b^	−	.	0	−	−	−	−
	Yes	−0.292	0.360	.658	1	0.417	0.747	0.368	1.513
Microdontia	No	−15.271	521.989	.001	1	.977	2.333E-7	0.001	0^c^
	Yes	0^b^	−	−	0	.	.	.	−
Respondents									
H/O trauma of respondents	Yes	-0.576	0.565	1.037	1	.309	.562	0.186	1.703
	No	0^b^	−	−	0	.	−	−	−
Fusion	No	-1.712	0.471	13.23	1	0.000	0.180	0.072	0.454
	Yes	0^b^	−	−	0	−	−	−	−
Gemination	No	-15.805	413.352	0.001	1	0.969	1.368E-7	0.000	0^c^
	Yes	0b	−	−	0	−	−	−	−
Nonsyndromic supernumerary teeth	No	1.887	0.335	31.65	1	0.000	0.151	0.078	0.292
	Yes	0b	−	−	0	−	−	−	−
Microdontia	No	−1.966	0.629	9.770	1	0.002	.140	.041	.480
	Yes	0^b^	−	−	0	−	−	−	−
Religion									
Hindu		−31.214	739.925	0.002	1	0.966	2.780 E-14	0.000	0^b^
Islam		20.314	0.704	831.912	1	.000	6.642E8	1.670E8	2.641E9
Christian		16.722	1.202	193.507	1	.000	1.829E7	1734127.067	1.930E8
Buddhist		19.615	0.000	−	1	−	3.301E8	3.301E8	3.301E8
Sikh		0^c^	−	−	0	−	−	−	−

Similarly, an association of consanguinity with the nonsyndromic occurrence of multiple dental anomalies like supernumerary, congenitally missing teeth, fusion, microdontia in a 12-year-old female was observed.*16* Nonsyndromic supernumerary teeth have also shown a true correlation with consanguineous marriages in studies conducted in Saudi Arabia^17^ and Lebanon,^18^ hence supporting the present study. Contrary to the above, 2,611 preschool children, aged 2–6-year-old were evaluated in a study conducted in Taiwan for the prevalence of congenital dental anomalies like hypodontia, hyperdontia, fusion, gemination, etc., but no association with consanguinity could be ascertained.^19^ A similar study was carried out in Turkey in 2012, where 1,149 children, aged 2–5 years were evaluated for dental anomalies namely fusion, gemination, microdontia, hyperdontia, but again no relation could be established with consanguinity.^20^ Similarly in Indore (India), 1123 subjects were investigated, 34.28% of the subjects presented with dental developmental anomalies like ectopic eruption (7.93%), hypodontia (4.19%), hyperdontia (2.40%), microdontia (2.58%), talons cusp (0.97%) though no relation with consanguinity was ascertained.^21^

## LIMITATIONS

Since the study was a household survey with such a large sample, so a radiographic examination was not possible. Being a cross-sectional study, it had the inherent drawbacks in the study design, hence have no idea about the etiology, period prevalence, and incidence rate.

## CONCLUSION

The study was able to deduce a true correlation of nonsyndromic supernumerary teeth, fusion, and microdontia in parents with consanguineous marriage and their children. Further, a collaboration between dental professionals and geneticists is needed to explore the underlying genetic factors, to create a pedigree chart of the family and to impart premarital counseling, education, and awareness amongst patients that not only medical conditions but dental conditions too have an association with consanguinity. Early diagnosis of the patients based on a pedigree chart can improve the treatment outcome.

### Why is it Important for Pediatric Dentist

- Dentists can play an active role along with the geneticist in premarital counseling and patient education.
- This voluntary action will help to create awareness amongst patients that not only medical conditions but dental conditions too have an association with consanguinity.
- Early diagnosis of the patients based on a pedigree chart can improve the treatment outcome.
